# Genome-wide identification and expression profiling reveal tissue-specific expression and differentially-regulated genes involved in gibberellin metabolism between Williams banana and its dwarf mutant

**DOI:** 10.1186/s12870-016-0809-1

**Published:** 2016-05-27

**Authors:** Jingjing Chen, Jianghui Xie, Yajie Duan, Huigang Hu, Yulin Hu, Weiming Li

**Affiliations:** Key Laboratory of Tropical Fruit Biology, Ministry of Agriculture, South Subtropical Crops Research Institute, Chinese Academy of Tropical Agricultural Sciences, Zhanjiang, 524091 China; National Field Genebank for Tropical Fruit (Zhanjiang), South Subtropical Crops Research Institute, Chinese Academy of Tropical Agricultural Sciences, Zhanjiang, 524091 China

**Keywords:** Gibberellins, Banana, GA oxidase genes, Early GA biosynthesis genes, Expression patterns, Tissue specificity

## Abstract

**Background:**

Dwarfism is one of the most valuable traits in banana breeding because semi-dwarf cultivars show good resistance to damage by wind and rain. Moreover, these cultivars present advantages of convenient cultivation, management, and so on. We obtained a dwarf mutant ‘8818-1’ through EMS (ethyl methane sulphonate) mutagenesis of Williams banana 8818 (*Musa* spp. AAA group). Our research have shown that gibberellins (GAs) content in 8818-1 false stems was significantly lower than that in its parent 8818 and the dwarf type of 8818-1 could be restored by application of exogenous GA_3_. Although GA exerts important impacts on the 8818-1 dwarf type, our understanding of the regulation of GA metabolism during banana dwarf mutant development remains limited.

**Results:**

Genome-wide screening revealed 36 candidate GA metabolism genes were systematically identified for the first time; these genes included 3 *MaCPS*, 2 *MaKS*, 1 *MaKO*, 2 *MaKAO*, 10 *MaGA20ox*, 4 *MaGA3ox*, and 14 *MaGA2ox* genes. Phylogenetic tree and conserved protein domain analyses showed sequence conservation and divergence. GA metabolism genes exhibited tissue-specific expression patterns. Early GA biosynthesis genes were constitutively expressed but presented differential regulation in different tissues in Williams banana. GA oxidase family genes were mainly transcribed in young fruits, thus suggesting that young fruits were the most active tissue involved in GA metabolism, followed by leaves, bracts, and finally approximately mature fruits. Expression patterns between 8818 and 8818-1 revealed that *MaGA20ox4*, *MaGA20ox5*, and *MaGA20ox7* of the *MaGA20ox* gene family and *MaGA2ox7*, *MaGA2ox12*, and *MaGA2ox14* of the *MaGA2ox* gene family exhibited significant differential expression and high-expression levels in false stems. These genes are likely to be responsible for the regulation of GAs content in 8818-1 false stems.

**Conclusion:**

Overall, phylogenetic evolution, tissue specificity and differential expression analyses of GA metabolism genes can provide a better understanding of GA-regulated development in banana. The present results revealed that *MaGA20ox4*, *MaGA20ox5*, *MaGA20ox7*, *MaGA2ox7*, *MaGA2ox12*, and *MaGA2ox14* were the main genes regulating GA content difference between 8818 and 8818-1. All of these genes may perform important functions in the developmental processes of banana, but each gene may perform different functions in different tissues or during different developmental stages.

**Electronic supplementary material:**

The online version of this article (doi:10.1186/s12870-016-0809-1) contains supplementary material, which is available to authorized users.

## Background

Height of cultivated banana generally exceeds 2 m, and its false stem is easily broken in typhoon-frequented areas. The stocky build of dwarf banana varieties can resist typhoon damage to a certain extent and offers the advantages of cultivation convenience, field management, labor savings, close planting, and so on. The dwarf mutant is a useful material for excavating and researching dwarf-related genes. Identification and utilization of banana dwarf-related genes are of considerable significance in breeding dwarf banana varieties.

We obtained the dwarf mutant ‘8818-1’ through EMS mutagenesis of Williams banana 8818. The stature of the 8818-1 false stem is approximately 1.7 m. Williams 8818-1 is stronger, bears shorter fruits, and presents dwarf characteristics in comparison with its parent, 8818. Previous studies reveal that hormone-deficient dwarf mutants can be restored by application of the corresponding exogenous active hormones in which the active hormone biosynthesis pathway is inhibited or blocked [1–3]. While dwarf mutants may become hormone-insensitive because of problems in hormone signal absorption, transfer, metabolic regulation genes, application of the corresponding exogenous active hormone can‘t restore the dwarf type [2, 4]. Total GAs contents in the false stem of Williams banana dwarf mutant 8818-1 are significantly lower than those in its parent 8818, and the plant stature of 8818-1 can be restored by application of exogenous active gibberellin GA_3_. We thus speculate that 8818-1 may be a hormone-deficient dwarf mutant.

GAs perform fundamental functions in plant growth and development, participating in the regulation of numerous developmental processes, such as seed germination [5, 6], stem elongation [7], leaf stretching [8], flower induction [9], and fruit-setting [10, 11]. Reduction of active GAs content causes plants to exhibit the dwarf phenotype. GA biosynthesis pathway is well elucidated in model plants, and their related mutants have been isolated [12]. GAs are biosynthesized from geranyl diphosphate, a common C20 precursor for diterpenoids. Biosynthesis enzymes, including *ent*-copalyl diphosphate synthase (CPS), *ent*-kaurene synthase (KS), *ent*-kaurene oxidase (KO), *ent*-kaurenoic acid oxidase (KAO), GA 20-oxidase(GA20ox), GA 3-oxidase(GA3ox), and GA 2-oxidase(GA2ox) [12, 13], may be classified as terpene synthases (TPSs), including CPS and KS, cytochrome P450 monooxygenases (P450s), including KO and KAO, and 2-oxoglutarate–dependent dioxygenases (2ODDs), including GA20ox, GA3ox, and GA2ox.

CPS, KS, KO, and KAO enzymes involved in the early steps of the GA metabolism pathway are usually encoded by a single or few genes [14]. Their mutants display severe dwarfism and loss of fertility, which can be recovered after spraying with exogenous active GAs [15–19]. Although multiple homologous genes are present in numerous plants, only one of these genes participates in the GA metabolism pathway. For instance, the rice OsCPS and OsKS-like gene families consist of 3 and 11 members, respectively, but only OsCPS1 and OsKS1 are responsible for ent-kaurene biosynthesis [20].

GA20ox, GA3ox and GA2ox are three enzymes that catalyze later reactions in the GA biosynthesis pathway and belong to the 2OG-Fe (II) oxygenase superfamily. In numerous plant species, the enzymes are independently encoded by different gene families [12, 21], thus accounting for certain functional redundancy, as well as tissue specificity [22]. The loss of function of these GA oxidase genes (except for GA2ox) in plants can generate a dwarf phenotype, which is restored by the application of exogenous GA [22–25]. For instance, the well-known Green Revolution Gene, *sd-1*, is generated from loss of function in *OsGA20ox2* of rice [26]. By contrast, GA2ox decreases levels of active GAs in plants, and overexpression of *GA2ox* genes can lead to dwarf types [27, 28].

GA metabolism genes have been identified in fungi, bacteria [29], Arabidopsis [30–35], rice [3], maize [36], soybean [21], pumpkin [37], pea [38, 39], cucumber [40], grapevine [41], *Brachypodium* [42], bread wheat [42], and *Salvia miltiorrhiza* [43], among others. Most publications focus on the systematic evolutionary analysis of the GA oxidase gene family in these plants, and gene functional research on individual pathway member from several plants has been conducted.

Previous results have shown that rice (*Oryza sativa*) possesses 8 *GA20ox*, 2 *GA3ox*, and 11 *GA2ox* genes; Arabidopsis possesses 5 *GA20ox*, 4 *GA3ox*, and 8 *GA2ox* genes; and soybean (*Glycine max*) contains 8 *GA20ox*, 6 *GA3ox*, and 10 *GA2ox* genes [21]. These GA oxidase genes exhibit a unique expression pattern and perform distinct developmental functions in different organs, tissues, and developmental stages of plants [21, 22, 33, 35, 44]. For instance, *AtGA3ox1* and *AtGA3ox2* are responsible for bioactive GA biosynthesis during vegetative growth, while *AtGA3ox1*, *AtGA3ox3*, and *AtGA3ox4* are important for the development of reproductive organs [22, 33]. Among the 5 *AtGA20ox* genes, *AtGA20ox1*, *AtGA20ox2*, and *AtGA20ox3* are the dominant paralogs [35]. *AtGA20ox3* is functionally redundant with *AtGA20ox1* and *AtGA20ox2*, whereas *AtGA20ox4* and *AtGA20ox5* perform minor roles in most developmental stages [35]. Differential expression and distinct developmental functions have also been observed in rice [3, 21, 45, 46]. Moreover, the transcription levels of several, but not all, GA metabolism genes are under feedback control [30, 47–49]. Control includes inhibition of the expression levels of several GA20ox and GA3ox genes, as well as activation of several GA2ox genes [12, 22, 27].

Banana A genome sequencing was completed in 2012 [50], but related information on GA metabolism in banana is limited. The numbers of GA metabolism genes in the banana A genome and their phylogenetic evolution, function, tissue specificity, and timing of expression have neither been verified nor explored. To understand the distribution and system evolution of GA metabolism genes in banana A genome, we searched all GA metabolism genes in The Banana Genome Hub and the National Center for Biotechnology Information (NCBI). Preliminary analyses of the system evolution of these genes have laid the foundation for research on banana GA metabolism genes. The expression levels of GA metabolism genes in Williams banana 8818 and 8818-1 and the principal genes regulating GAs content remain unknown. To elucidate possible causes of the 8818-1 dwarf phenotype, we analyzed tissue specificity and compared the gene expression differences in seven kinds of genes encoding early GA biosynthesis genes and GA oxidase genes between 8818 and 8818-1. These results improve our current understanding of the GA metabolism pathway in banana and contribute to research in other closely related species with significant agricultural importance.

## Results

### GAs content analysis and exogenous GA_3_ application treatment

**Fig. 1:**
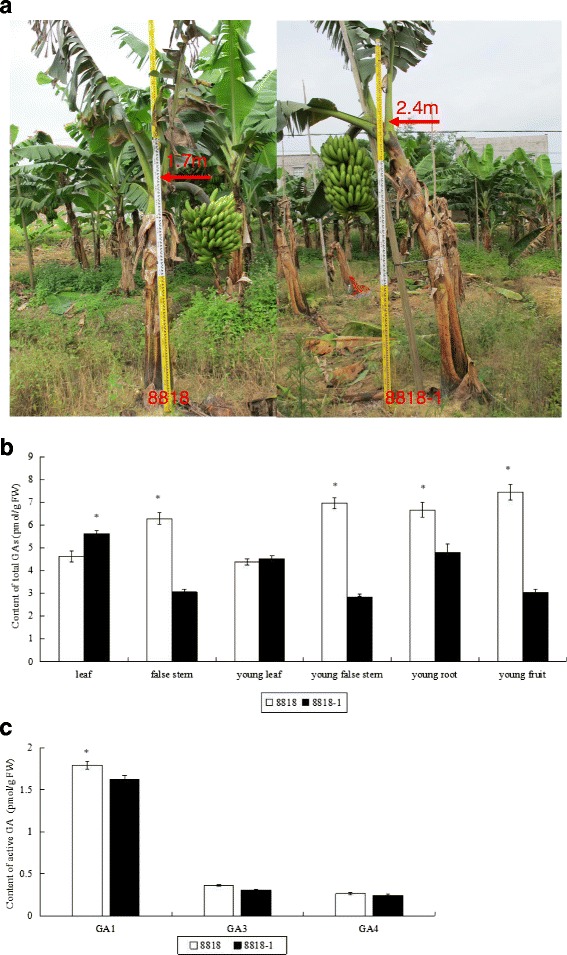
Phenotypes and gibberellins levels of banana mutant 8818-1 and its wide type(8818). **a** Comparison of the plant height between 8818 and 8881-1 in the harvest period. **b** Total GAs contents between 8818 and 8818-1 in different tissues at different ages. **c** Active GAs (GA_1_, GA_3_ and GA_4_) contents in false stems of 8818 and 8818-1. Significant difference of total GAs contents for each tissue and active GA contents for each GA between 8818 and 8818-1 estimated by *t*-test was reported on the graphics (*p*-value < 0.05). Stars (*) indicate significant differences of total GAs content between the same organ of 8818 and 8818-1 (**b**) or between the same active GA of 8818 and 8818-1 (**c**)

In the field, the adult 8818-1 plant presented stronger, shorter false stems and shorter fruits in comparison with the parent 8818 (Fig. 1a). Total GAs content was determined in different tissues of Williams 8818 and its mutant, 8818-1. The results are shown in Fig. 1b. In addition to that in leaves, the total GAs contents in most tissues of 8818-1 were lower than those in 8818 during different developmental stages. Total GAs contents of false stems during the young and adult development stages in 8818 were 113 % and 145 % higher than those in 8818-1, respectively. Total GAs contents of young fruits and roots in 8818 were also significantly higher than those in 8818-1. Either during adulthood or the seedling stage, the total GAs content of 8818-1 false stems was significantly lower than that of 8818. GAs have several forms and many of them are inactive and intermediates, and only few are active forms, namely GA_1_, GA_3_ and GA_4_. So contents of GA_1_, GA_3_ and GA_4_ were determined in false stems of 8818 and 8818-1 (Fig. 1c). The results showed that GA_1_ was the highest content active GA and the three kinds of active GAs content of false stems in 8818-1 were all lower than those in 8818. Among them the difference of GA_1_ content between 8818 and 8818-1 was significant. False stems are closely related to plant stature; therefore, 8818-1 is significantly shorter than 8818, which may be due to a decline in GAs content in the former, especially GA_1_ content.

**Fig. 2:**
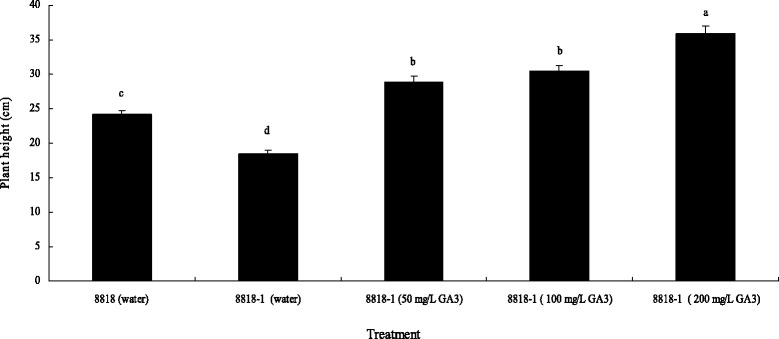
Effect of exogenous GA_3_ treatments on plant height of 8818-1 with different concentrations. Each value was the mean of ten biological replicates with the standard error indicated and evaluated by Duncan’s test (*p*-value < 0.05). Means labeled by the same letter are not significantly different

Exogenous GA_3_ (50, 100, and 200 mg/L) application was conducted on 8818-1; in this experiment, water was used as a control. Results suggested that treatment with all three concentrations could restore the plant height of 8818-1 to 8818 levels or even higher (Fig. 2). GA_3_ exerted a dose-dependent effect on 8818-1; the higher the concentration, the more rapidly the false stems elongated within the scope of 50–200 mg/L GA_3_.

Considering the results of GAs content determination and plant height recovery, we can speculate that the dwarfism of 8818-1 may be caused by reduction of GAs content in false stems.

### Isolation of putative GA metabolism genes in banana

To identify the genes encoding seven kinds of GA metabolism enzymes in the banana A genome, we screened all available banana amino acid sequences in the Banana Genome Hub and NCBI. The banana A genome was sequenced and published in 2012. The sequenced genotype is a doubled-haploid (2*n* = 22, 1C = 523 Mb) from the *Musa acuminata* (A genome) subsp. Malaccencis DH-Pahang [50]. Three *CPS*-like genes (*MaCPS*1-3), 2 *KS*-like genes (*MaKS*1-2), 2 *KAO-*like genes (*MaKAO*1-2), 1 *KO-*like gene (*MaKO1*), 10 *GA20ox-*like genes (*MaGA20ox* 1–10), 5 *GA3ox-*like genes (*MaGA3ox1-3*), and 15 *GA2ox-*like genes (*MaGA2ox1-15*) were searched. In the banana A genome, 38 candidate genes were distributed across all 11 banana chromosomes and 1 random chromosome (Table 1; Additional file 1). We named the genes according to their position in the chromosome.

**Table 1:** Gibberellin metabolism genes and their homologs in banana A genome

Enzyme	Gene name	Acession number in NCBI	Entry name	Chromosome location
CPS	*MaCPS1*	XP_009414733.1	GSMUA_Achr8T31500_001	chr8:33156487..33157292 (− strand)
	*MaCPS2*	XP_009414734.1	GSMUA_Achr8T31510_001	chr8:33158109..33162457 (− strand)
	*MaCPS3*	XP_009415635.1	GSMUA_Achr8T31530_001	chr8:33168336..33172673 (− strand)
KS	*MaKS1*	XP_009381749.1	GSMUA_Achr10T20910_001	chr10:26761414..26763280 (+ strand)
	*MaKS2*	XP_009381751.1	GSMUA_Achr10T20940_001	chr10:26771313..26772514 (+ strand)
KO	*MaKO*	XP_009403115.1	SMUA_Achr6T00910_001	chr6:620666..628430 (+ strand)
KAO	*MaKAO1*	XP_009392783	GSMUA_Achr3T27540_001	chr3:27071455..27081269 (+ strand)
	*MaKAO2*	XP_009420467	GSMUA_Achr10T06490_001	chr10:16816835..16818498 (− strand)
GA20ox	*MaGA20ox1*	XP_009380434.1	GSMUA_Achr2T01010_001	chr2:5960401..5961658 (+ strand)
	*MaGA20ox2*	XP_009396824.1	GSMUA_Achr4T16380_001	chr4:14661621..14663603 (− strand)
	*MaGA20ox3*	XP_009406147.1	GSMUA_Achr6T25910_001	chr6:26881996..26883403 (+ strand)
	*MaGA20ox4*	XP_009407673.1	GSMUA_Achr7T08230_001	chr7:6140804..6142227 (+ strand)
	*MaGA20ox5*	XP_009407673.1	GSMUA_Achr7T08240_001	chr7:6146847..6148188 (+ strand)
	*MaGA20ox6*	XP_009414611.1	GSMUA_Achr8T19120_001	chr8:24064366..24065656 (− strand)
	*MaGA20ox7*	XP_009413747.1	GSMUA_Achr8T32560_001	chr8:33911692..33913414 (− strand)
	*MaGA20ox8*	XP_009383569.1	GSMUA_Achr11T11840_001	chr11:20062818..20064276 (+ strand)
	*MaGA20ox9*	XP_009385199.1	GSMUA_Achr11T18740_001	chr11:10722740..10724748 (− strand)
	*MaGA20ox10*	XP_009387900.1	GSMUA_AchrUn_randomT21840_001	chrUn_random:106671560..106672879 (− strand)
GA3ox	*MaGA3ox1*	XP_009390400.1	GSMUA_Achr1T03100_001	chr1:2492380..2493414 (+ strand)
	*MaGA3ox2*	XP_009396646.1	GSMUA_Achr4T08970_001	chr4:6533960..6536897 (+ strand)
	*MaGA3ox3*	XP_009400517.1	GSMUA_Achr5T09790_001	chr5:7004255..7005466 (+ strand)
	*MaGA3ox4*	XP_009409327.1	GSMUA_Achr7T13240_001	chr7:10639164..10640374 (− strand)
	*MaGA3ox5*	XP_009385827.1	GSMUA_AchrUn_randomT03870_001	chrUn_random:17581786..17582964 (+strand)
GA2ox	*MaGA2ox1*	XP_009394604.1	GSMUA_Achr3T31410_001	chr3:29737137..29738643 (+ strand)
	*MaGA2ox2*	XP_009395077.1	GSMUA_Achr4T00800_001	chr4:691523..692733 (+ strand)
	*MaGA2ox3*	XP_009396510.1	GSMUA_Achr4T15110_001	chr4:11391241..11393337 (+ strand)
	*MaGA2ox4*	XP_009405644.1	GSMUA_Achr6T21950_001	chr6:18633392..18636939 (+ strand)
	*MaGA2ox5*	XP_009406244.1	GSMUA_Achr6T26900_001	chr6:27521888..27523063 (− strand)
	*MaGA2ox6*	XP_009409401.1	GSMUA_Achr7T13930_001	chr7:11167366..11168849 (− strand)
	*MaGA2ox7*	XP_009412952.1	GSMUA_Achr8T03660_001	chr8:2497885..2502247 (− strand)
	*MaGA2ox8*	XP_009415245.1	GSMUA_Achr8T27270_001	chr8:30495418..30496693 (+ strand)
	*MaGA2ox9*	XP_009416515.1	GSMUA_Achr9T06460_001	chr9:4127576..4129282 (− strand)
	*MaGA2ox10*	XP_009417251.1	GSMUA_Achr9T11880_001	chr9:7697712..7699360 (+ strand)
	*MaGA2ox11*	XP_009418345.1	GSMUA_Achr9T21260_001	chr9:26308679..26310286 (+ strand)
	*MaGA2ox12*	XP_009421396.1	GSMUA_Achr10T13090_001	chr10:21898631..21900169 (− strand)
	*MaGA2ox13*	XP_009380496.1	GSMUA_Achr10T21600_001	chr10:27150831..27152767 (− strand)
	*MaGA2ox14*	XP_009383703.1	GSMUA_Achr11T14320_001	chr11:15359030..15362781 (− strand)
	*MaGA2ox15*	XP_009386085.1	GSMUA_AchrUn_randomT06450_001	chrUn_random:26248924..26250412 (−strand)

#### Early GA biosynthesis genes

We searched two CPS-like complete cDNA sequences (MaCPS2 and MaCPS3) and one CPS-like (MaCPS1, GSMUA_Achr8T31500_001) fragment sequence in the Banana Genome Hub and then searched the complete cDNA sequence of MaCPS1 in NCBI. The three genes were all located in chromosome 8. MaCPS1 presented 98.54 and 84.27 % identities with MaCPS2 and MaCPS3, respectively, and MaCPS 1, 2, and 3 showed 45.38, 44.82, and 48.71 % identities with OsCPS (Os02g0278700). In NCBI, Blast analysis revealed that MaCPS 1, 2, and 3 showed the highest similarity to the CPS of *Phoenix dactylifera*, as well as 74, 72, and 76 % identities with PdCPS, respectively.

Two MaKS-like complete cDNA sequences were searched in NCBI. Both sequences were located on chromosome 10 and shared 62.70 % identity. In NCBI, MaKS-like revealed the highest similarity to KS of *Elaeis guineensis* (77 and 78 % identity) but shared only 41.6 and 31.52 % identity with OsKS (Os04g0611800). In NCBI and the Banana Genome Hub, we found only one MaKO-like gene, which was located in chromosome 6, sharing the highest similarity to KO of *Phoenix dactylifera* (77 %) and 62.50 % identity with OsKO/CYP701A(D35) (Os06g0570100). Two MaKAO-like genes were located in chromosomes 3 and 10 shared 75.16 % identity with each other, maximum similarities to *KAO* of *Phoenix dactylifera* (79 and 76 %), and 62.33 and 67.38 % identity with OsKAO/CYP88A5 (Os06g0110000), respectively.

#### GA oxidase genes (GA20ox, GA2ox, and GA3ox)

GA20ox, GA3ox, and GA2ox are three enzymes that catalyze later reactions in the GA biosynthesis pathway. These enzymes belong to the 2OG-Fe (II) oxygenase superfamily and are encoded by a multigene family [12]. Ten GA20ox-like genes were found in the banana A genome; in comparison, 5 and 8 copies of GA20ox genes have been reported in Arabidopsis and rice, respectively [21, 43]. Ten GA20ox-like genes were located on chromosomes 2, 4, 6, 7, 8, and 11 (Additional file 1). In rice, *OsGA20ox2* is reported as the rice Green Revolution Gene and is previously known as Semi-Dwarf1 (SD1) [51]; loss of function of *OsGA20ox2* can generate the dwarf phenotype. The deduced amino acid sequence of banana MaGA20ox2 (GSMUA_Achr4T16380_001) showed the highest homology with OsGA20ox2/SD1 (68.65 % identity); by comparison, MaGA20ox4 (GSMUA_Achr7T08230_001) revealed only 40.76 % identity with the gene.

Five GA3ox-like genes were searched in the Banana Genome Hub. However, four GA3ox genes were searched in NCBI. Four GA3ox-like genes in the Banana Genome Hub respectively matched four GA3ox genes searched by BLAST in NCBI. Meanwhile, MaGA3ox1 (GSMUA Achr1P03100) showed 100 % identity with banana GA20ox genes by blast X in NCBI. Phylogenetic analysis also revealed that MaGA3ox1 was grouped as a single clade and possessed a distant genetic relationship with the GA3ox genes of rice, maize, and Arabidopsis. Therefore, the annotation of GSMUA Achr1P03100 in the Banana Genome Hub should be revised. In comparison, two and four copies of GA3ox genes have been reported in Arabidopsis and rice, respectively [21, 43]. Genetic evidence from the *d18* mutant (defective in *OsGA3ox2*) proves that *OsGA3ox2* is essential and that loss of function of *OsGA3ox2*/D18 can generate the dwarf phenotype. Four GA3ox-like genes (MaGA3ox2-5) showed 59.66, 57.26, 56.85, and 56.67 % identities with this gene.

Fifteen GA2ox-like genes were searched in the banana A genome. By comparison, 7 and 11 copies of GA2ox genes have been reported in Arabidopsis and rice, respectively [21, 43]. Fifteen GA2ox-like genes were distributed to the rest of the chromosomes, except for chromosomes 1, 2, 5. However, BLAST X in NCBI revealed that MaGA2ox2 (GSMUA_Achr4T00800_001) shared 100 % identity with the *Musa acuminata* probable 2-oxoglutarate-dependent dioxygenase gene. Phylogenetic analysis of GA oxidase genes showed that MaGA2ox2 presented a distant genetic relationship with other GA2ox genes. Thus, we speculate that MaGA2ox2 belongs to the 2OG-Fe (II) oxygenase superfamily and not the GA2ox family.

### Analyses of phylogenetic tree and conserved protein domains of GA metabolism genes in banana and other plants

#### Early GA biosynthesis genes

**Fig. 3:**
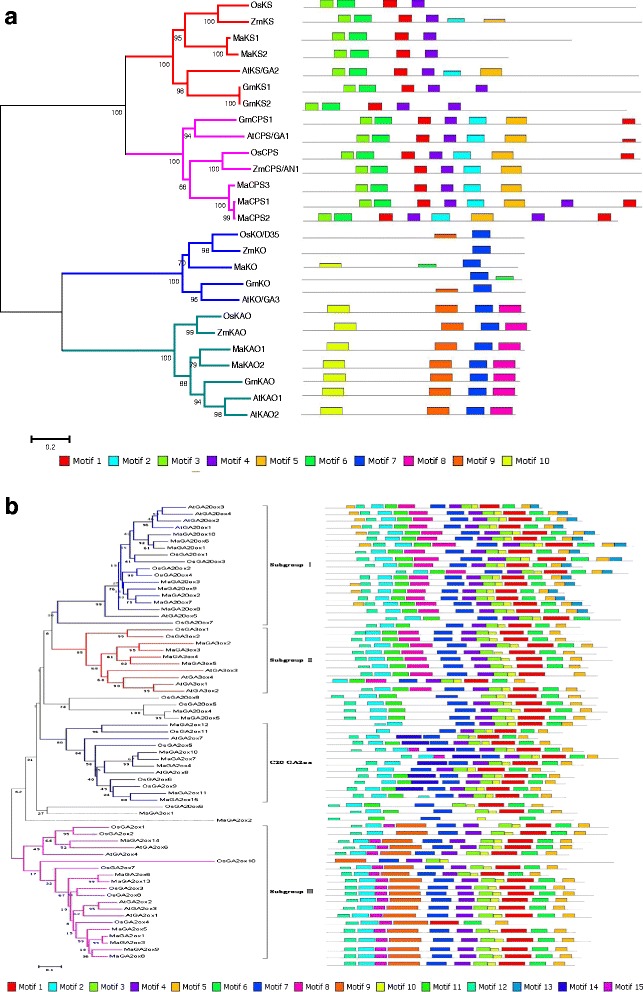
Analysis of phylogenetic relationships and conserved protein motifs among GA metabolism genes. **a** Early GA biosynthesis genes (MaCPS, MaKS, MaKO and MaKAO). **b** GA oxidase genes (MaGA20ox, MaGA3ox, and MaGA2ox). Ma, *Musa acuminata*; At, *Arabidopsis thaliana*; Os, *Oryza sativa*; Gm, *Glycine max*; Zm, Zea mays. The accession numbers of protein sequences cited in this study are in Additional file 2

Phylogenetic analysis of diterpene cyclases (CPS and KS) and Cyt P450 monooxygenases (KO and KAO) (Fig. 3a) amino acid sequences from banana, rice, maize, soybean, and Arabidopsis (Additional file 2) revealed that CPS, KS, KO, and KAO proteins could be divided into monocot and dicot groups. This finding is consistent with banana, rice, and maize which are monocot plants. The monocot group was subdivided into two subgroups; rice and maize were grouped in the same clade, whereas banana presented a distant genetic relationship with rice and maize among monocot plants. In NCBI, BLAST analysis showed that *Elaeis guineensis* and *Phoenix dactylifera* shared the highest similarity to banana. Phylogenetic analysis revealed that three CPS-like proteins were highly similar and grouped in the same clade; moreover, two KS-like and KAO-like which belonged to Cyt P450 monooxygenases were grouped in the same clade (Fig. 3a).

Analysis of conserved domains (Fig. 3a) revealed that all CPS possessed motifs 1, 2, 3, 4, 5, and 6 in common, whereas KS owned motifs 1, 3, 4, and 6. We thus speculate that protein domains 1, 3, 4, and 6 are specific to the diterpene cyclases. CPS differed from KS by possessing conserved motifs 2 and 5. KAO only contained conserved motifs 7, 8, 9, and 10, which suggested evolutionary conservation. KO only possessed motif 7, which could be common in all Cyt P450-dependent monooxygenases.

#### GA oxidase genes

To identify the evolutionary relationships of the GA oxidase genes in banana, Arabidopsis, and rice, we constructed multiple sequence alignments based on the GA20ox, GA3ox, and GA2ox protein sequences of banana, Arabidopsis, and rice (Additional file 2). An evolutionary tree was established according to the alignment results by using the neighbor joining (NJ) method (Fig. 3b). Phylogenetic analysis showed that most GA oxidase genes could be mainly separated into four subgroups (I, II, III, and C20 GA2ox). Subgroups I, II, and III clearly corresponded to differences among the functions of GA20ox, GA3ox, and GA2ox. GA20ox and GA3ox can promote the production of active GA, whereas GA2ox inactivates GA, thereby regulating GA content in plants [21].

The phylogenetic tree revealed that the GA oxidases of rice, Arabidopsis, and banana were more similar to their respective homologs within each subgroup than to each other. This finding indicated that expansion of GA oxidase genes occurred early in the evolution of this protein family. GA3ox belonged to a smaller gene family than GA20ox and GA2ox. Four, two, and four copies of GA3ox genes were discovered in Arabidopsis, rice, and banana, respectively. By contrast, 5, 8, and 10 copies of GA20ox genes and 7, 11, and 14 copies of GA2ox genes were discovered in Arabidopsis, rice, and banana, respectively. This finding indicated that the GA3ox gene family was more conserved than the GA20ox and GA2ox families. Moreover GA20ox and GA3ox were separated by a relatively small distance (Fig. 3b), whereas GA2ox was located farther from these genes.

Several homologous sequences of GA20ox and GA2ox showed low sequence identity, and certain branches disclosed a pronounced divergence and did not cluster together. Six MaGA2ox genes (MaGA2ox4, MaGA2ox7, MaGA2ox10, MaGA2ox11, MaGA2ox12, and MaGA2ox15) didn’t appear in subgroups I, II, and III. These genes constituted a separate branch with OsGA2ox5, OsGA2ox6, OsGA2ox9, OsGA2ox11, AtGA2ox7, and AtGA2ox8, showing less similarity to other GA2ox proteins. Previous results have verified that OsGA2ox5, OsGA2ox9, OsGA2ox6, OsGA2ox11, AtGA2ox7, and AtGA2ox8 belong to C20 GA2ox [21, 45]. Thus, six MaGA2ox genes may also belong to C20 GA2ox.

C20 GA2ox was found to hydroxylate C20-GA precursors (converting GA12 and GA53 to GA110 and GA97, respectively) but not C19-GAs, thus decreasing active GA levels [21, 34]. For instance, OsGA2ox9 have been verified to inactivate bioactive GA_1_, thereby repressing cell growth [44], similar to members in subgroup III. Overexpression of wild-type or modified C20 GA2ox in rice can produce a semi-dwarf type, increase root systems, and higher tiller numbers [45]. C20 GA2ox split from C19 GA2ox in the phylogenetic tree (Fig. 3b), but the key functional regions of coding sequences in GA oxidase were less variable (Fig. 3b). C20 GA2ox exists not only in rice, Arabidopsis, and banana but also in other plants, such as SoGA2ox3 from spinach [45] and GmGA2ox4 from soybean [21]. In banana, six C20 GA2oxs are found, which suggests that C20 GA2ox may be widespread in plant GA metabolism.

Moreover, several GA oxidases, such as OsGA20ox5, OsGA20ox6, OsGA20ox7, OsGA20ox8, MaGA20ox4, and MaGA20ox5, didn’t appeared in the four subgroups and were not clustered together with GA20ox, which implies GA20ox genes may have more complicated evolution.

Protein domains 2, 3, 4, 5, 6, 7, and 12 were in common in most GA20ox, GA2ox, and GA3ox genes. We found that protein domain 13 was unique to subgroup I and subgroup III exclusively possessed protein domains 9 and 15. Protein domain 14 was exclusively contained by C20-GA2ox, and subgroup II possessed no special protein domain, suggesting greater conservation in evolution. Protein domain 8 only existed in subgroups I and II; this domain was lacking in subgroup III and C20-GA2ox. C20-GA2ox didn’t possess protein domain 10 which existed in subgroups I, II, and III. These special motifs may account for the function difference.

In three kinds of GA oxidase genes, the numbers of genes of GA20ox and GA2ox were greater than that of GA3ox and these genes possessed considerably longer branches in the phylogenetic trees. These findings indicated that GA20ox and GA2ox evolved more rapidly than GA3ox. GA20ox and GA2ox demonstrated more dynamic evolutionary routes, thereby resulting in greater functional redundancy. In addition, more copies of GA20ox and GA2ox could cause relaxed selective pressure or loosened constraints in the evolution process. Subgroups I, II, and III contained both monocot and dicot proteins. This evolutionary relationship suggests that every subgroup of GA20ox/GA3ox/GA2ox proteins may perform homologous functions crossing between monocot and dicot plants [21, 28, 52].

### Tissue specificity analysis of GA metabolism genes in Williams banana

**Fig. 4:**
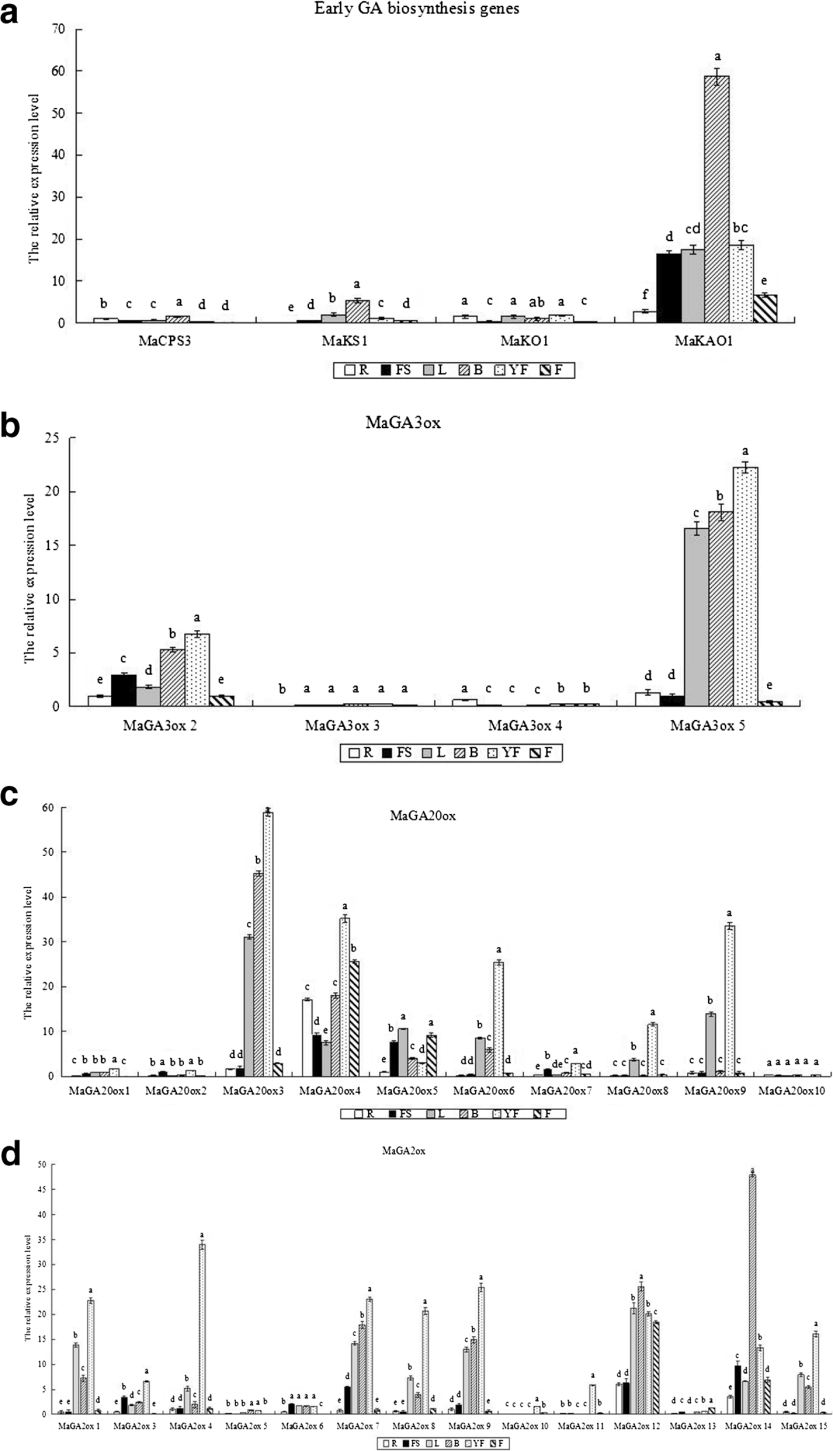
Tissue-specific expression of the GA metabolism genes in various tissues of banana dwarf mutant 8818-1. Total RNAs were isolated from leaves (L), bracts (B), false stems (FS), roots (R), young fruits (YF), and approximately mature fruits (F) in growth and development period, and qRT-PCR was conducted. Relative expression value was calculated using ‘*actin*’ as a reference by 2^-ΔΔCt^ method. Expression level of *MaCPS3*, *MaGA3ox2*, *MaGA20ox5* and *MaGA2ox4* in the root were respectively defined as 1 in a, b, c and d. Fold change of the target gene in every tissue, normalized to *actin* and relative to the expression of *MaCPS3*, *MaGA3ox2*, *MaGA20ox5*, *MaGA2ox4* in the root respectively in a, b, c and d, was calculated for each sample using the 2^-ΔΔCt^ method. The CT value of each gene was the average of three technical replicates with the standard error indicated. Significant difference of relative expression value in different tissues of the same target gene estimated by Duncan’s test was reported on the graphics (*p*-value < 0.05). Means labeled by the same letter are not significantly different

Quantitative real-time polymerase chain reaction (qRT-PCR) analysis revealed that the isolated GA metabolism genes were expressed at different levels in various tissues of Williams banana 8818-1 (Fig. 4).

*MaCPS3*, *MaKS1*, *MaKO1*, and *MaKAO1* were broadly expressed at different levels in all tested tissues of Williams banana 8818-1, including leaves, roots, false stems, bracts, young fruits, and approximately mature fruits (Fig. 4a). The expression level of *MaKAO1* gene in different tissues was generally higher than those of the three other genes in the corresponding tissues. The expression level of *MaKAO1* was the highest in the bract, followed by leaves, false stems, and young fruits. The highest gene expression levels of *MaCPS3* and *MaKS1* were observed in bracts, whereas the highest level of *MaKO1* expression was found in young fruits. As a whole, expression level of *MaKAO1* in all tissues was the highest among the early GA biosynthesis genes tested, while difference among other three genes expression levels in all tissues was small, thus suggesting that *MaKAO1* might play an important regulating role in transcription level in GA biosynthesis of the banana.

Analysis of four *GA3ox-*like genes (*MaGA3ox2*, *MaGA3ox3*, *MaGA3ox4*, and *MaGA3ox5*) revealed that they were expressed at different levels in six tissues (Fig. 4b). *MaGA3ox2* expression levels were higher in young fruits and bracts but lower in approximately mature fruits. Compared with *MaGA3ox4* and *MaGA3ox5*, *MaGA3ox3* and *MaGA3ox4* were present at lower expression levels. The relative expression level of *MaGA3ox3* in young fruits was the highest among six tissues, but the relative expression value remained below 0.3, similar to the relative expression value of *MaGA3ox4* (<0.3) in all tissues not including roots. *MaGA3ox5* was strongly expressed in young fruits, bracts, and leaves by 22-fold, 18-fold, and 16-fold, respectively, compared with that of *MaGA3ox2* in roots. While *MaGA3ox5* was weakly expressed in the roots, false stems, and approximately mature fruits. The *MaGA3ox2* and *MaGA3ox5* of four *GA3ox-*like genes may be the key genes regulating GA content in the normal development of banana. However, different genes perform different functions in various tissues.

*MaGA20ox1*, *2*, and *10* showed relatively low expression and revealed less obvious tissue specificity in vegetative tissues. By contrast, other genes exhibited high expression in several tissues at least (Fig. 4c). *MaGA20ox3* showed relatively high expression in leaves, bracts, and young fruits but low expression in roots, false stems, and approximately mature fruits. The expression level of *MaGA20ox4* was relatively high in all tested plant tissues, presenting the highest expression in young fruits and the lowest expression in leaves. *MaGA20ox5* was prominently expressed in leaves, false stems, and approximately mature fruits and lowly expressed in roots. *MaGA20ox6* was also expressed in all tissues, and showed extremely high levels in young fruits and extremely low levels in roots. These results reveal obvious tissue specificity.

*MaGA20ox8* and *MaGA20ox9* expression levels were relatively similar, showing evident tissue specificity, particularly high expression in young fruits and low expression in roots, false stems, and approximately mature fruits. *MaGA20ox7* expression was higher than those of *MaGA20ox1*, *2* and *10* but lower than those of abundant genes, such as *MaGA20ox3*, *MaGA20ox4*, and so on. Tissue specificity among these genes was evident. In general, young fruits contained abundant genes, except *MaGA20ox5* and *MaGA20ox10*, and other *MaGA20ox* genes all demonstrated maximum expression levels in young fruits.

Fourteen *MaGA2ox* genes could generally be divided into two categories. Genes included in the first category were strongly expressed in most tissues and expressed differently in most tissues. This group included *MaGA2ox1*, *MaGA2ox3*, *MaGA2ox4*, *MaGA2ox7*, *MaGA2ox8*, *MaGA2ox12*, *MaGA2ox14*, and *MaGA2ox15*. Genes in the second category were weakly expressed and slightly high expression in individual tissues. This group included *MaGA2ox5*, *MaGA2ox6*, *MaGA2ox10*, *MaGA2ox11*, and *MaGA2ox1*3. *MaGA2ox12* demonstrated the highest expression level in roots, followed *MaGA2ox14*; other *MaGA2ox* genes were weakly expressed.

In false stems, *MaGA2ox14* was the most abundant gene, although *MaGA2ox12*, *MaGA2ox7*, *MaGA2ox3*, and *MaGA2ox6* were also strongly expressed. Other *MaGA2ox* genes were weakly expressed. *MaGA2ox12* showed the highest expression in leaves, followed by *MaGA2ox7*, *MaGA2ox1*, *MaGA2ox15*, and *MaGA2ox8*; by contrast, *MaGA2ox5*, *MaGA2ox10*, *MaGA2ox11*, and *MaGA2ox13* were lowly expressed in this tissue. In bracts, the top three most abundantly expressed genes included *MaGA2ox14*, *MaGA2ox12*, and *MaGA2ox7*. Numbers of high-expression genes in young fruits exceeded those in other tissues. *MaGA2ox4* was the most high- expression gene, followed by *MaGA2ox1*, *MaGA2ox7*, *MaGA2ox8*, *MaGA2ox12*, *MaGA2ox15*, *MaGA2ox14*, and *MaGA2ox6*.

In approximately mature fruits, highly expressed *MaGA2ox* genes were few. *MaGA2ox12* was the most strongly expressed gene, followed by *MaGA2ox14*; all other genes were weakly expressed. The analysis described above reveals that genes expressed strongly in most tissues, namely, *MaGA2ox14*, *MaGA2ox12*, and *MaGA2ox7*, and so on, may be the key genes regulating GA content. Higher expression levels of genes were found in false stems, bracts, leaves, especially in young fruits, and such expression was of considerable significance for regulating GA contents in these tissues. *MaGA2ox* gene family expression patterns in different tissues in 8818-1 may explain changes in the morphological characteristics of 8818-1, to a certain extent, such as dwarf false stems and shorter fruits, in comparison with those of 8818.

### Differential expression analysis of GA metabolism genes in the false stem of Williams banana 8818 and 8818-1

**Fig. 5:**
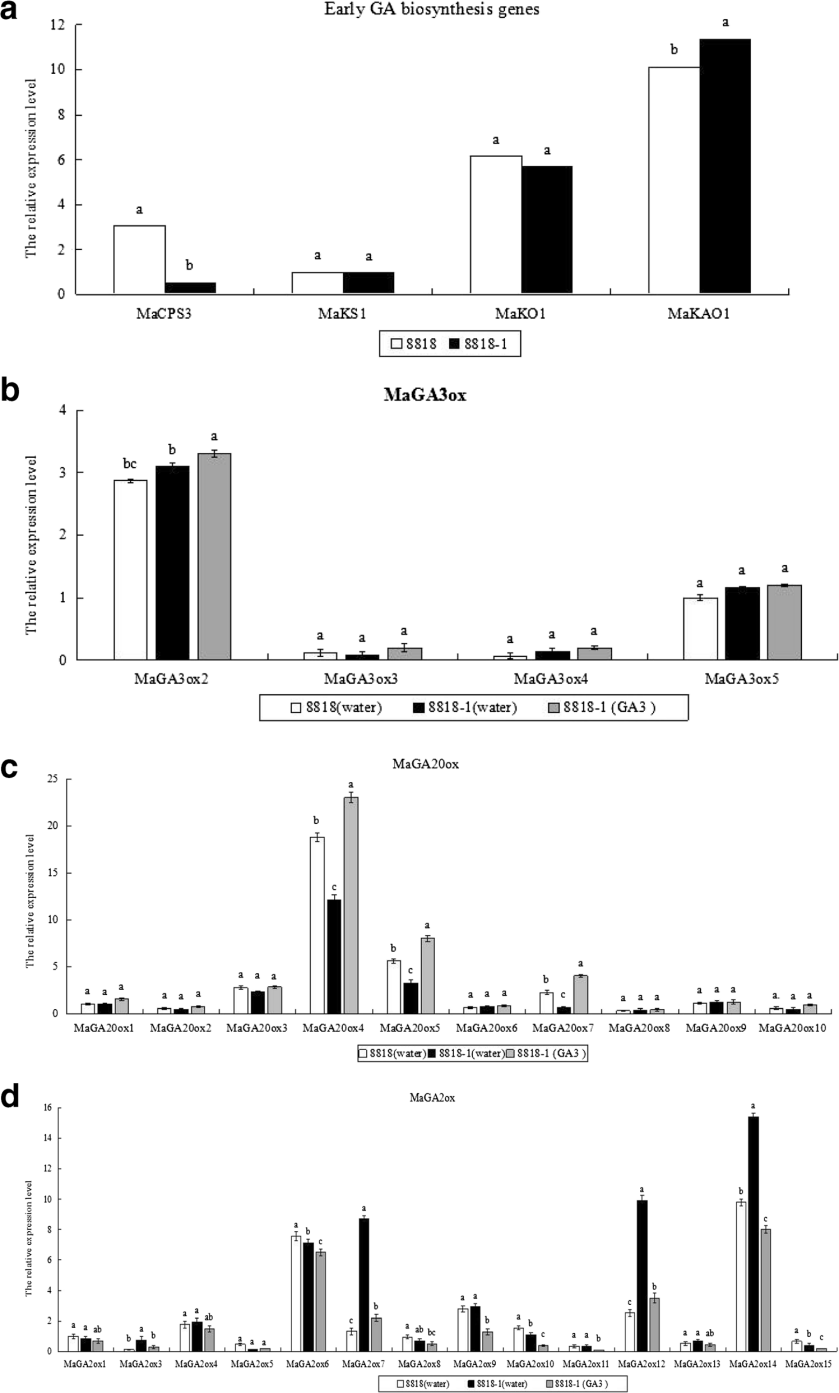
Expression analysis of GA metabolism genes in false stems of Williams banana 8818 and its mutant 8818-1. **a** Early GA biosynthesis genes (*MaCPS3*, *MaKS1*, *MaKO1*, *MaKAO1*). **b** *MaGA3ox*. **c** *MaGA20ox*. **d** *MaGA2ox*. Total RNAs were isolated from 8818 to 8818-1 false stems when the plant had grown to eight leaves and qRT-PCR was conducted. Relative expression value was calculated using ‘*actin*’ by 2^-ΔΔCt^ method. Expression level of *MaKS1*, *MaGA20ox1*, *MaGA3ox5*, *MaGA2ox1* in 8818 were respectively defined as 1 in a, b, c and d. The CT value of each gene was the average of three technical replicates with the standard error indicated. Significant difference of relative expression value in the same target gene estimated by Duncan’s test was reported on the graphics (*p*-value < 0.05). Means labeled by the same letter are not significantly different

The differential expression of *MaCPS3*, *MaKS1*, *MaKO1*, and *MaKAO1* in false stems of Williams banana 8818 and its mutant 8818-1 was analyzed (Fig. 5a). *MaKAO1* was the most highly expressed gene among the four early GA biosynthesis genes in the false stems of 8818 and 8818-1. Compared with 8818, *MaKAO1* was more highly expressed in 8818-1. By contrast, 8818-1 showed lower expression levels of *MaCPS3* than 8818, and the difference observed was significant. *MaKS1* and *MaKO1* did’t show significantly different expression levels between 8818 and 8818-1.

*MaGA3ox2* and *MaGA3ox5* of the four *GA3ox-*like genes showed higher expression levels than *MaGA3ox3* and *MaGA3ox4* in false stems (Fig. 5b). The expression levels of the four genes in 8818 and 8818-1 showed differences that were not significant. After exogenous GA_3_ application on 8818-1, expression levels of four genes were all more or less higher than those in 8818-1 treated with water, had no significant difference between them. GA3ox gene expression may not be the main cause of total GAs and active GA contents differences in the false stems of 8818 and 8818-1.

Analysis of the expression levels of ten *MaGA20ox-*like genes in the false stems of Williams banana 8818 and its mutant 8818-1 revealed that *MaGA20ox4* was the most prominently expressed GA20ox family member, being expressed 18-fold higher in 8818 than *MaGA20ox1* in 8818 and 3-fold higher than *MaGA20ox5* in 8818 (*MaGA20ox5* expression is after *MaGA20ox4*) (Fig. 5c). *MaGA20ox4* expression in 8818-1 was significantly lower than that in 8818, and only 65 % of the expression value in 8818. Moreover, the expression levels of *MaGA20ox3*, *MaGA20ox5*, and *MaGA20ox7* were higher than those of other *MaGA20ox* genes. The *MaGA20ox5 and MaGA20ox7* expression levels in 8818 was 1.7-fold and 3-fold higher than those in 8818-1, respectively. The expression level of *MaGA20ox3* in 8818 was higher than that in 8818-1, but no significant difference between them. Other *MaGA20ox* genes, such as *MaGA20ox1*, *MaGA20ox2*, *MaGA20ox6*, *MaGA20ox8*, *MaGA20ox9*, and *MaGA20ox10*, showed similar expression levels in 8818 and 8818-1 with no significant difference.

After exogenous GA_3_ application on 8818-1, the results revealed the expression levels of *MaGA20ox4*, *MaGA20ox5*, and *MaGA20ox7* increased significantly than those in 8818-1 treated with water. Although expression levels of other *MaGA20ox* genes were more or less increased after exogenous GA_3_ application, they had no significant difference. These results suggested that *MaGA20ox4*, *MaGA20ox5*, and *MaGA20ox7* in *MaGA20ox* gene family were the main genes induced by exogenous active GA in false stems of banana.

Overall, expression levels of *MaGA20ox4*, *MaGA20ox5*, and *MaGA20ox7* in 8818-1 were significantly lower than those in 8818, meanwhile, these three genes were dramatically induced in 8818-1 false stems. Therefore we speculate that *MaGA20ox4*, *MaGA20ox5*, and *MaGA20ox7*, may play important regulating roles in GA synthesis of banana false stems, and their expression differences may cause differences of total GAs and active GA contents between 8818 and 8818-1.

As shown in Fig. 5d, *MaGA2ox6*, *MaGA2ox7*, *MaGA2ox12*, and *MaGA2ox14* exhibited higher expression levels among 14 *MaGA20ox*-like gene family members. The expression levels of *MaGA2ox7*, *MaGA2ox12*, and *MaGA2ox14* in 8818-1 were prominently lower than those in 8818, respectively only 15 %, 25 %, and 63 % of the expression levels in 8818; *MaGA2ox6* expression level was not significantly different between 8818 and 8818-1. *MaGA2ox3*, *MaGA2ox5*, *MaGA2ox10*, and *MaGA2ox15* demonstrated lower expression levels than the above four genes (*MaGA2ox6*, *MaGA2ox7*, *MaGA2ox12*, and *MaGA2ox14*) in false stems with significant difference between 8818 and 8818-1. *MaGA2ox1*, *MaGA2ox4*, *MaGA2ox8*, *MaGA2ox9*, *MaGA2ox11*, and *MaGA2ox13* also showed lower expression levels than the above four genes (*MaGA2ox6*, *MaGA2ox7*, *MaGA2ox12*, and *MaGA2ox14*) in false stems, but no significant differences between 8818 and 8818-1 were observed.

In 8818-1 false stems, most genes of *MaGA2ox* gene family were inhibited by exogenous GA_3_ treatment. Expression of *MaGA2ox3*, *MaGA2ox7*, *MaGA2ox9*, *MaGA2ox10*, *MaGA2ox11*, *MaGA2ox12*, *MaGA2ox14* and *MaGA2ox15* were inhibited and had significant difference in 8818-1 false stems between GA_3_ and water treatment. *MaGA2ox9* and *MaGA2ox11* were significantly inhibited by GA_3_ treatment, but they showed no significant difference between 8818 and 8818-1, so they may be not the key regulating genes. *MaGA2ox3*, *MaGA2ox10* and *MaGA2ox15* were significantly inhibited by GA_3_ treatment and their expression levels had significant differences between 8818 and 8818-1, but they showed lower expression levels, perhaps not the main regulating genes. *MaGA2ox7*, *MaGA2ox12*, and *MaGA2ox14* not only had higher expression levels, but also their expression levels in 8818-1 were prominently lower than those in 8818, probably play important regulating roles in GA synthesis in banana false stems.

## Discussion

### Phylogenetic analysis of GA metabolism enzyme genes in banana

#### Early GA biosynthesis genes

In banana A genome, genes corresponding to enzymes involved in the early stages of GA biosynthesis pathway have several homologous sequences, which is also reported in other plants [15–19]. Although multiple CPS-like, KS-like, KAO-like, and KO-like genes are present, only one of these genes is a GA metabolism enzyme in most plants [20]. In Arabidopsis, the CPS, KS, and KO enzymes involved in GA biosynthesis are encoded by single genes [53]. The rice genome contains two genes that encode a functional CPS, but only OsCPS1 has been proven to participate in GA biosynthesis by mutant studies; OsCPS2 is involved in the biosynthesis of diterpene phytoalexins [3]. Prisic and Xu [18] compared OsCPS1ent, OsCPS2ent, and OsCPSsyn sequences with An1/ZmCPS1ent from maize (*Zea mays*) and found that OsCPS1ent was more similar to An1/ZmCPS1ent (64 % identity) than to either one of its paralogs (44 %). Three CPS-like genes were screened in banana A genome; MaCPS1 and MaCPS2 possess particularly high homology, whereas compared with MaCPS1 and MaCPS2, MaCPS3 shares higher identity with OsCPS1. MaCPS3 probably involves in GA synthesis, whereas MaCPS1 and MaCPS2 may involve in the biosynthesis of phytoalexins in banana. However, this hypothesis needs further investigation. Although KS and KO each possess two homologous genes with low identity, both enzymes probably perform different functions, similar to multiple KS-like and KO-like sequences existing in the rice genome. However, KS and KO in GA biosynthesis are also encoded by single genes [3, 53].

#### Late GA biosynthesis and inactivation genes

We studied the evolutionary properties of GA oxidase gene family in rice, Arabidopsis, and banana to elucidate the expansion patterns of banana GA oxidase genes after species split. According to their phylogeny, most of GA oxidase genes were mainly divided into four subgroups (I, II, III, and C20 GA2ox), thus reflecting functional classifications corresponding to GA20ox, GA3ox, GA2ox, and C20 GA2ox. These results are consistent with a study on rice, Arabidopsis, and soybean by Han and Zhu [21]. Previous results suggest that 16, 21, 17 and 24 GA oxidase genes are discovered in Arabidopsis, rice, grapevine, and soybean, respectively [3, 21, 41]. In comparison, banana A genome contains up to 28 GA oxidase genes, thereby suggesting that GA oxidases in banana are more divergent than those in Arabidopsis and rice. Given the common conservative protein domain observed, these four subgroups perform similar functions.

Few sequence changes occur in GA3ox in Arabidopsis, rice, and banana, thus implying that GA3ox may be under greater pressure and more conserved in comparison with other GA oxidases. However, more copies of GA20ox and GA2ox exist, and the sequences of these genes are more divergent. GA2ox gene family is divided into two subgroups, namely, subgroups III and C20 GA2ox, and several GA oxidase genes are not included in the four subgroups. These genes, including OsGA20ox5, OsGA20ox6, OsGA20ox7, OsGA20ox8, MaGA20ox4, and MaGA20ox5, evolved from GA20ox. These results suggest that GA20ox and GA2ox likely undergo a more dynamic evolutionary route, resulting in greater functional redundancy, especially in banana. More copies of GA20ox and GA2ox probably cause relaxed selective pressure or fewer constraints during evolution. On the other hand, GA20ox and GA3ox are separated by a relatively small distance. Together with the positive regulation of GA20ox and GA3ox in GA biosynthesis, the results indicate that genes essential for survival are preferentially produced. Although the GA2ox gene family possesses more divergent sequences, subgroups III and C20 GA2ox may share similar functions. Overexpression of C20 GA2ox in wild-type is found to cause semi-dwarfism by inactivating C20 GA precursors [34]. These findings imply that GA2ox genes perform important functions in a variety of approaches.

### Differential expression of GA metabolism genes in dwarf and normal banana cultivars

The tissue-specific expression patterns of GA metabolism genes suggest that each member of them may perform different physiological functions. Precise regulation of GA content appears to be critical in plant normal development. Early GA biosynthesis genes (CPS, KS, KO, and KAO) are often single-copy genes in most plants. Although several homologous genes exist, only one gene is proven by mutant studies to participate in the GA metabolic pathway [3, 53]. As these genes are expressed in the early GA synthesis phase, extreme dwarf plants easily occur with mutation. However, if only the expression level of one gene is changed, dwarfism will probably not occur, because control of the GA biosynthesis pathway is mainly exerted by GA oxidases through feedback loop mechanisms [54] and localization of their expression to limited tissues. Early GA biosynthesis genes (*MaCPS3*, *MaKS1*, *MaKO1*, and *MaKAO1*) are broadly expressed in all tested tissues of Williams banana 8818-1. *MaCPS3* is the first gene in GA synthesis pathway and the expression level of *MaCPS3* is lower in 8818-1 in comparison with 8818. While, *MaKAO1* shows higher expression in 8818-1. Therefore, the expression difference of *MaCPS3* may not be enough to decrease GAs and active GA contents in 8818-1.

In GA3ox gene family, *MaGA3ox2* and *MaGA3ox5* present higher expression levels and evident tissue specificity. Thus, the two genes likely perform important functions in different tissues during certain development stages in banana. The expression levels of the four genes in 8818 and 8818-1 show statistically insignificant differences. Therefore, changes in the expression levels of these genes may not lead to GA content reduction in false stems of 8881-1.

The gene families of GA20ox and GA2ox include more family members than GA3ox gene family. Previous research suggests that functional diversification of surviving duplicated genes is an important feature of long-term evolution of polyploids [55]. This finding implies that the GA20ox and GA2ox gene families probably perform diverse and important functions in banana. Most of the evidences point to the dynamic regulation of GA biosynthesis through regulation of 2-ODD genes in contrast to terpene cyclase and cytochrome P450 genes in earlier stages of the pathway [56]. Alteration of the expression levels of GA oxidases has been proven to be successful in controlling plant stature in most plants and often affects several traits [41]. Loss of function of *OsGA20ox2* can generate dwarf phenotypes [51]. Overexpression of *PtaGA2ox1* in poplar produce a short and stout phenotype [57], whereas a dwarf plum hybrid shows enhanced expression of *PslGA2ox* [58].

Except for *MaGA20ox1*, *2*, and *10*, other *MaGA20ox* genes show high expression levels in several tissues, thus revealing evident tissue specificity. *MaGA20ox* genes present the highest expression levels in young fruits, possibly because the young fruit is in a critical period of growth and development and needs more active GA to promote fruit elongation. By contrast, high-expression genes in roots are few, indicating that the root in detected period is not the main area of GA synthesis. *MaGA20ox4* and *MaGA20ox5* were the most highly expressed genes among the detected tissues, meanwhile, they show significantly different expression levels between 8818 and 8818-1 false stems. These observations suggest that the two *GA20ox* genes not only perform important functions in the development of whole plant organs but also may be the core genes regulating GA content in banana false stems. It is noteworthy that MaGA20ox4 and MaGA20ox5 are excluded from the four subgroups, along with OsGA20ox5, OsGA20ox6, and OsGA20ox8, and don’t cluster together with other GA20ox. Recent research has also demonstrated that *OsGA20ox5* and *OsGA20ox6* are expressed in callus, root, leaf, and flower in rice [21]. These results imply that this type of genes have important functions in the plant development. Moreover, some researchers have postulated that multiple functionalization of GA20ox can lead to sequence divergence [21].

On the basis of the distances in the phylogenetic tree, MaGA2ox genes are divided into subgroups III (C19 GA2ox) and C20 GA2ox. According to the expression levels in detected tissues, *MaGA2ox* genes are divided into high-expression and low-expression genes. Subgroups III and C20 GA2ox both include high-expression and low-expression genes with similar numbers, maybe the two subgroups both play important roles in banana growth and development. In rice, improvements in plant architecture, such as semi-dwarfism, increased root systems, and higher tiller numbers, can be induced by overexpression of C20 GA2ox or modified C20 GA2ox [45]. C20 GA2ox provides an alternative mechanism for controlling endogenous GA levels.

Tissue specificity analysis reveals that more high-expression GA oxidase genes are present in young fruits and false stems. In young fruits in particular, the genes are of considerable significance for regulating activity of active GA content. Multiple genes regulation may greatly aid in maintaining GA contents at the appropriate levels and satisfying the needs of cells and organs at different developmental stages. GA concentrations can be regulated precisely at this stage and transported to tissues requiring further elongation.

Bioactive hormone concentrations are regulated at the level of hormone synthesis through controlled inactivation [27, 43]. Down-regulation of certain GA20ox and GA3ox genes (also known as negative feedback regulation) and up-regulation of several GA2ox genes (also known as positive forward regulation) can control bioactive GA content. *MaGA2ox7*, *MaGA2ox12*, and *MaGA2ox14* of the *MaGA2ox* gene family present significantly different expressions with high-expression levels in 8818 and 8818-1 false stems, and are markedly inhibited by exogenous GA_3_. *MaGA20ox4*, *MaGA20ox5*, and *MaGA20ox7* also present significantly different expressions with high- expression levels in 8818 and 8818-1 false stems, and are markedly induced by exogenous GA_3._ The low expression of *MaGA20ox* genes (*MaGA20ox4*, *MaGA20ox5*, and *MaGA20ox7*) in 8818-1 and the high expression of *MaGA2ox* genes (*MaGA2ox7*, *MaGA2ox12*, and *MaGA2ox14*) in 8818-1 can both lead to reducing active GA content. To a certain extent, the different expression between 8828 and 8818-1 and the expression patterns of *MaGA20ox* and *MaGA2ox* genes in different tissues of 8818-1 can explain the changes in morphological characteristics of 8818-1, such as dwarf false stems and shorter fruits, in comparison with 8818. These genes may be the key genes regulating GA content in Williams banana false stems.

## Conclusions

In this research, we screened all banana GA metabolism genes in A genome, as well as analyzed their homologous evolutionary relationship and identified the tissue specificity of these genes. These analyses may help elucidate metabolism genes in other monocotyledonous herbs.

We found 36 GA metabolism genes and gene features and phylogenetic analysis showed their conservation and divergence in Williams banana. Moreover, our study found that the most high-expression GA oxidase genes existed in young fruits. This finding demonstrates that young fruits are the most active areas of GA metabolism, which contributes to fruit length regulation. We investigated the key genes regulating GA content in banana false stem by expression difference analysis not only between 8818 and its dwarf mutant 8818-1, but also in 8818-1 treated with water and GA_3_. GA oxidase genes, including *MaGA2ox7*, *MaGA2ox12*, *MaGA2ox14*, *MaGA20ox4*, *MaGA20ox5*, and *MaGA20ox7*, may perform key regulating functions. Differences in the expression levels of these genes in terms of GA content in 8818-1 false stems are lower than those in 8818, thus explaining the semi-dwarf phenotype of the 8818-1 mutant. Based on tissue specificity and expression level differences, identifying important GA metabolism enzyme genes can help us obtain new banana cultivars with stocky features but comparable fruit lengths through genetic engineering. The present results will also benefit research on other closely related species with significant agricultural importance.

## Methods

### Plant materials

Banana cultivars used in this experiment were Williams banana 8818 (*Musa acuminata* AAA group, cv. Cavendish, var. Williams) and its mutant, 8818-1. Williams 8818 is a kind of widely grown commercial cultivar. Our research team immersed shoot apex of Williams 8818 used 0.4 % EMS for 3 h, then screened and obtained a dwarf mutant strain by the field planting, named 8818-1. Williams 8818 and its mutant 8818-1 were grown in the Plant Resources Nursery of South Subtropical Crops Institute, CATAS, Zhanjiang. We selected the leaves(L), roots(R), false stems(FS) of Williams banana 8818-1 when the plants had grown to the eight-leaf stage as materials of tissue specificity analysis; the bracts(B), young fruits(YF), and approximately mature fruits(F) of 8818-1 were also collected as materials of tissue specificity analysis. All of the samples were frozen in liquid nitrogen after sampling, and then stored at −80 °C for further use. All assessments were conducted with three biological replicates.

### Determinations of GAs content

Young leaves, young roots and young false stems of Williams banana 8818 and 8818-1 when the plants had grown to eight-leaf stage and false stems, young fruits, leaves when the plants were fully grown were selected as materials for total GAs content determination. For active GA (GA_1_, GA_3_ and GA_4_) contents determination, we only selected young false stems of 8818 and 8818-1 grown to eight leaves as materials. Total GAs and active GA contents of Williams banana 8818 and 8818-1 were determined by using an enzyme-linked immunosorbent assay kit (Rapidbio, USA), according to the manufacturer’s instructions and as described by Yang and Guo [59]. Each tissue was sampled from three individual plants as replicates, and the determination was conducted three times for each sample.

### GA_3_ treatment and plant growth measurement

To determine GA_3_ promotion on plant growth, William 8818-1 plants grown to the eight-leaf stage were sprayed with GA_3_ solutions (50, 100 and 200 mg/L); Williams banana 8818 and 8818-1 were sprayed with water as controls until the leaves and false stems were fully wetted (10 mL/pot), followed by spraying once every five days. Ten pots were used for each treatment as replicates. Plant height was measured after 30 days.

The false stems of William banana 8818 and 8818-1 grown to the eight-leaf stage were collected as materials for differential expression analysis of GA metabolism genes. Meanwhile, the false stems of 8818-1 in eight-leaf stage sprayed with 200 mg/L GA_3_ solutions were collected after 4 h. All materials were frozen in liquid nitrogen after sampling, and then stored at −80 °C for further use. All assessments were conducted with three biological replicates.

### Sequence retrieval, alignment, and phylogenetic analysis

All sequences were obtained from four databases: TAIR (the Arabidopsis Information Resource, http://www.arabidopsis.org/), Rice Genome Annotation Project Database (http://rice.plantbiology.msu.edu/), The Banana Genome Hub (http://banana-genome.cirad.fr/blast), and the NCBI (http://www.ncbi.nlm.nih.gov/). Sequence searches of banana GA metabolism genes were conducted in Banana A genome and NCBI. We only selected sequences with full-length cDNA sequence and excluded sequences with only cDNA fragment. All of GA metabolism genes are listed in Table 1.

To determine sequence similarity with other species, BLAST search was performed against NCBI by using the protein sequence of banana GA metabolism genes by Blast P. Protein sequence alignment of early GA biosynthesis genes from banana, Arabidopsis, rice, and soybean, and GA oxidase genes from banana, rice and Arabidopsis were analyzed respectively by Clustalx1.83 and MEGA5.0 software through NJ method for phylogenetic tree construction. Parameters included the bootstrap method for testing of phylogeny, 1000 bootstrap replications, random seeds and Poisson’s model. Conserved protein motifs were analyzed by using the online software, MEME (http://meme-suite.org/tools/meme) [60], and parameters setting were as follows: maximum number of conserved motifs, 15; and size range, 6–200 amino acid residues.

For early GA biosynthesis genes (CPS, KS, KO, and KAO) owned multiple sequences, we selected the one with the highest homology with corresponding rice genes for tissue specificity and differential expression analyses. OsCPS (Os02g027870), OsKS (Os04g0611800), OsKO (Os06g0570100), and OsKAO (Os06g0110000) genes have been proven to participate in GA synthesis pathway. Therefore, we selected and aligned these genes with banana early GA biosynthesis genes (MaCPS, MaKS, MaKO, and MaKAO) by using DNASTAR software.

### qRT–PCR expression analysis

Frozen tissues were ground in liquid nitrogen using a mortar and pestle. Total RNAs were extracted by using Quick RNA isolation Kit (Hua Yue Yang, Beijing, China) according to the manufacturer's protocol. Potentially contaminating DNA was eliminated by treatment with DNAse I digestion and using a RNAse-free kit (Hua Yue Yang, Beijing, China). The DNA-free total RNA was used as the template for reverse transcription. First-strand cDNA was synthesized from 1 μg of total RNA by using the PrimeScript™ RT reagent Kit with gDNA Eraser (Takara Bio. Inc., Dalian, China). The above cDNA was diluted 10-fold and then used as templates for the PCR reaction.

Primers for quantitative reverse transcription PCR (qRT-PCR) were designed by using Primer Premier 5.0 software (Premier, Canada) and synthesized by Sangon Biotech Co. Ltd. (Shanghai, China). Banana *actin* gene (GenBank Accession Number: AB022041) was selected as a reference. All primers are shown Additional file 3. Primer specificity was validated by melting profiles and showed a single product specific melting temperature. Quantitative reverse transcription PCR was performed on a LightCycler®480 Real-Time PCR System (Roche, Basel, Switzerland) using a SYBR Green-based PCR assay and three technical replicates were used per sample for the qRT-PCR experiments. The CT value of each gene was the average of three technical replicates.

Each reaction mixture was 20 μL, containing 6 μL of diluted first-strand cDNAs (250 nM of each primer) and 10 μL of TransStart Tip SYBR Green master mix (TransGen Biotech, Beijing, China). Amplification procedure was set as follows: 95 °C for 5 min, followed by 40 cycles of 95 °C for 10 s, 58 °C for 20 s, and 72 °C for 25 s, then 95 °C for 5 s, 65 °C for 1 min, and 97 °C for fluorescence collection in 96-well optical reaction plates.

Expression levels of the tested genes were determined by CT values and calculated by 2^-ΔΔCt^ method [61]. The data were presented as the fold change in gene expression normalized to reference gene ‘*actin*’ and relative to the setting control. The relative expression value was analyzed using equation, where 2^-ΔΔCt^ = 2^(CT, Target- CT, Actin)Tissue x -(CT, Target-CT, Actin)Setting control^. Tissue x is any tissue and Setting control represents the 1 × expression of the target gene normalized to *actin*.

### Statistical analysis

All measurements were repeated three times. All data were subjected to analysis of variance according to the model for a completely randomized design by using SPSS software (SPSS Inc., Chicago, IL, USA). Differences between two sample evaluated by *t*-test at the 0.05 level and Differences among three or more samples were evaluated by Duncan’s test at the 0.05 level.

## Additional files

Additional file 1: Figure S1. Chromosomal locations of 38 banana candidate genes on the banana A genome. (PDF 61 kb) Additional file 2: Table S1. The accession numbers of protein sequences cited in this study. (PDF 45 kb) Additional file 3: Table S2. Primer sequences of the reference gene and GA metabolism genes for qRT-PCR in this study. (PDF 71 kb)

## Abbreviations

2ODDs: 2-oxoglutarate–dependent dioxygenases
CPS: ent-copalyl diphosphate synthase
EMS: ethyl methane sulphonate
GA20ox: GA20-oxidase
GA2ox: GA2-oxidase
GA3ox: GA3-oxidase
GAs: gibberellins
KAO: ent-kaurenoic acid oxidase
KO: ent-kaurene oxidase
KS: ent-kaurene synthase
NCBI: national center for biotechnology information
qRT-PCR: quantitative real-time polymerase chain reaction
TPSs: terpene synthases

## References

[1] Sun T-p (2000). Gibberellin signal transduction. Curr Opin Plant Biol.

[2] Yamaguchi S, Kamiya Y (2000). Gibberellin biosynthesis: its regulation by endogenous and environmental signals. Plant Cell Physiol.

[3] Sakamoto T, Miura K, Itoh H, Tatsumi T, Ueguchi-Tanaka M (2004). An overview of gibberellin metabolism enzyme genes and their related mutants in rice. Plant Physiol.

[4] Sun TP, Gubler F (2004). Molecular mechanism of gibberellin signaling in plants. Annu Rev Plant Biol.

[5] Tyler L, Thomas SG, Hu JH, Dill A, Alonso JM, Ecker JR (2004). Della proteins and gibberellin-regulated seed germination and floral development in Arabidopsis. Plant Physiol.

[6] Ayele BT, Ozga JA, Reinecke DM (2006). Regulation of GA biosynthesis genes during germination and young seedling growth of pea (*Pisum sativum* L.). J Plant Growth Regul.

[7] Junttila O, Jensen E, Pearce DW, Pharis RP (1992). Stimulation of shoot elongation in Salix pentandra by gibberellin GA3; activity appears to be dependent upon hydroxylation to GA1 via GA3. Plant Physiol.

[8] Hedden P, Proebsting WM (1999). Genetic analysis of gibberellin biosynthesis. Plant Physiol.

[9] Blazquez MA, Green R, Nilsson O, Sussman MR, Weigel D (1998). Gibberellins promote flowering of Arabidopsis by activating the LEAFY promoter. Plant Cell.

[10] Santes CM, Hedden P, Gaskin P, Garcia-Martinez JL (1995). Gibberellins and related compounds in young fruits of pea and their relationship to fruit-set. Phytochemistry.

[11] Serrani JC, Sanjuán R, Ruiz-Rivero O, Fos M, García-Martínez JL (2007). Gibberellin regulation of fruit set and growth in tomato. Plant Physiol.

[12] Hedden P, Phillips AL (2000). Gibberellin metabolism: new insights revealed by the genes. Trends Plant Sci.

[13] Yamaguchi S (2006). Gibberellin biosynthesis in Arabidopsis. Phytochem Rev.

[14] Hedden P, Thomas SG (2012). Gibberellin biosynthesis and its regulation. Biochem J.

[15] Silverstone AL, Chang C, Krol E, Sun T (1997). Developmental regulation of the gibberellin biosynthetic gene GA1 in *Arabidopsis thaliana*. Plant J.

[16] Helliwell CA, Poole A (1999). Arabidopsis ent-kaurene oxidase catalyzes three steps of gibberellin biosynthesis. Plant Physiol.

[17] Olszewski N, Sun TP, Gubler F (2002). Gibberellin signaling: biosynthesis, catabolism, and response pathways. Plant Cell.

[18] Prisic S, Xu M (2004). Rice contains two disparate ent-Copalyl diphosphate synthases with distinct metabolic functions. Plant Physiol.

[19] Xu M, Wilderman PR, Morrone D, Xu J, Roy A, Margis-Pinheiro M (2007). Functional characterization of the rice kaurene synthase-like gene family. Phytochemistry.

[20] Grennan AK (2006). Gibberellin metabolism enzymes in rice. Plant Physiol.

[21] Han F, Zhu B (2011). Evolutionary analysis of three gibberellin oxidase genes in rice, Arabidopsis, and soybean. Gene.

[22] Mitchum MG, Yamaguchi S, Hanada A, Kuwahara A, Yoshioka Y, Kato T (2006). Distinct and overlapping roles of two gibberellin 3-oxidases in Arabidopsis development. Plant J.

[23] Huang SS, Raman AS, Ream JE, Fujiwara H, Cerny RE, Brown SM (1998). Overexpression of 20-oxidase confers a gibberellin overproduction phenotype in Arabidopsis. Plant Physiol.

[24] Oikawa T, Koshioka M, Kojima K, Yoshida H, Kawata M (2004). A role of OsGA20ox1, encoding an isoform of gibberellin 20-oxidase, for regulation of plant stature in rice. Plant Mol Biol.

[25] Shan C, Mei ZL, Duan JL, Chen HY, Feng HF, Cai WM (2014). OsGA2ox5, a gibberellin metabolism enzyme, is involved in plant growth, the root gravity response and salt stress. PLoS One.

[26] Spielmeyer W, Ellis MH, Chandler PM (2002). Semidwarf (sd-1), “green Revolution”rice, contains a defective gibberellin 20-oxidase gene. Proc Natl Acad Sci U S A.

[27] Thomas SG, Phillips AL, Hedden P (1999). Molecular cloning and functional expression of gibberellin 2-oxidases, multifunctional enzymes involved in gibberellin deactivation. Proc Natl Acad Sci U S A.

[28] Schomburg FM, Bizzell CM, Lee DJ, Zeevaart JA, Amasino RM (2003). Overexpression of a novel class of gibberellin 2-oxidases decreases gibberellin levels and creates dwarf plants. Plant Cell.

[29] MacMillan J (2002). Occurrence of gibberellins in vascular plants, fungi, and bacteria. J Plant Growth Regul.

[30] Phillips AL, Ward DA, Uknes S, Appleford NE, Lange T, Huttly AK (1995). Isolation and expression of three gibberellin 20-oxidase cDNA clones from Arabidopsis. Plant Physiol.

[31] Helliwell CA, Chandler PM, Poole A, Dennis ES, Peacock WJ (2001). The CYP88A cytochrome P450, ent-kaurenoic acid oxidase, catalyzes three steps of the gibberellin biosynthesis pathway. Proc Natl Acad Sci U S A.

[32] Helliwell CA, Sullivan JA, Mould RM, Gray JC, Peacock WJ, Dennis ES (2001). A plastid envelope location of Arabidopsis ent-kaurene oxidase links the plastid and endoplasmic reticulum steps of the gibberellin biosynthesis pathway. Plant J.

[33] Hu J, Mitchum MG, Barnaby N, Ayele BT, Ogawa M, Nam E (2008). Potential sites of bioactive gibberellin production during reproductive growth in Arabidopsis. Plant Cell.

[34] Lee DJ, Zeevaart JA (2005). Molecular cloning of GA2-oxidase 3 from spinach and its ectopic expression in Nicotiana sylvestris. Plant Physiol.

[35] Plackett AR, Powers SJ, Fernandez-Garcia N, Urbanova T, Takebayashi Y, Seo M (2012). Analysis of the developmental roles of the Arabidopsis gibberellin 20-oxidases demonstrates that GA20ox1, −2, and −3 are the dominant paralogs. Plant Cell.

[36] Song J, Guo B, Song F, Peng H, Yao Y, Zhang Y (2011). Genome-wide identification of gibberellins metabolic enzyme genes and expression profiling analysis during seed germination in maize. Gene.

[37] Lange T, Kappler J, Fischer A, Frisse A, Padeffke T, Schmidtke S (2005). Gibberellin biosynthesis in developing pumpkin seedlings. Plant Physiol.

[38] Davidson SE, Swain SM, Reid JB (2005). Regulation of the early GA biosynthesis pathway in pea. Planta.

[39] Stavang JA, Lindgård B, Erntsen A, Lid SE, Moe R, Olsen JE (2005). Thermoperiodic stem elongation involves transcriptional regulation of gibberellin deactivation in pea. Plant Physiol.

[40] Lange MJP, Liebrandt A, Arnold L, Chmielewska SM, Felsberger A, Freier E (2013). Functional characterization of gibberellin oxidases from cucumber, *Cucumis sativus* L. Phytochemistry.

[41] Giacomelli L, Rota-Stabelli O, Masuero D, Acheampong AK, Moretto M, Caputi L (2013). Gibberellin metabolism in Vitis vinifera L. During bloom and fruit-set: functional characterization and evolution of grapevine gibberellin oxidases. J Exp Bot.

[42] Pearce S, Huttly AK, Prosser IM, Li YD, Vaughan SP, Gallova B (2015). Heterologous expression and transcript analysis of gibberellin biosynthetic genes of grasses reveals novel functionality in the GA3ox family. BMC Plant Biol.

[43] Du Q, Li CL, Li DQ, Lu SHF (2015). Genome-wide analysis, molecular cloning and expression profiling reveal tissue-specifically expressed, feedback-regulated, stress-responsive and alternatively spliced novel genes involved in gibberellin metabolism in Salvia Miltiorrhiza. BMC Genomics.

[44] Rieu I, Eriksson S, Powers SJ, Gong F, Griffiths J, Woolley L (2008). Genetic analysis reveals that C19-GA 2-Oxidation is a major gibberellin inactivation pathway in Arabidopsis. Plant Cell.

[45] Lo SF, Yang SY, Chen KT, Hsing YI, Zeevaart JA, Chen LJ (2008). A novel class of gibberellin 2-oxidases control semidwarfism, tillering, and root development in rice. Plant Cell.

[46] Plackett ARG, Thomas SG, Wilson ZA, Hedden P (2011). Gibberellin control of stamen development: a fertile field. Trends Plant Sci.

[47] Chiang HH, Hwang I, Goodman HM (1995). Isolation of the Arabidopsis GA4 locus. Plant Cell.

[48] Carrera E, Jackson SD, Prat S (1999). Feedback control and diurnal regulation of gibberellin 20-oxidase transcript levels in potato. Plant Physiol.

[49] Xu YL, Li L, Gage DA, Zeevaart JA (1999). Feedback regulation of GA5 expression and metabolic engineering of gibberellin levels in Arabidopsis. Plant Cell.

[50] D'Hont A, Denoeud F, Aury JM, Baurens FC, Carreel F, Garsmeur O (2012). The banana (Musa acuminata) genome and the evolution of monocotyledonous plants. Nature.

[51] Sasaki A, Ashikari M, Ueguchi-Tanaka M (2002). Green revolution :a mutant gibberellin synthesis gene in rice. Nature.

[52] Sakai M, Sakamoto T, Saito T, Matsuoka M, Tanaka H, Kobayashi M (2003). Expression of novel rice gibberellin 2-oxidase gene is under homeostatic regulation by biologically active gibberellins. J Plant Res.

[53] Yamaguchi S (2008). Gibberellin metabolism and its regulation. Annu Rev Plant Biol.

[54] Middleton AM, Úbeda-Tomás S, Griffiths J, Holman T, Hedden P, Thomas SG (2012). Mathematical modeling elucidates the role of transcriptional feedback in gibberellin signaling. Proc Natl Acad Sci U S A.

[55] Blanc G, Wolfe KH (2004). Widespread paleopolyploidy in model plant species inferred from age distributions of duplicate genes. Plant Cell.

[56] Huang Y, Yang W, Pei Z, Guo X, Liu D, Sun J (2012). The genes for gibberellin biosynthesis in wheat. Funct Integr Genomics.

[57] Busov VB, Meilan R, Pearce DW, Ma C, Rood SB, Strauss SH (2003). Activation tagging of a dominant gibberellin catabolism gene (GA 2-oxidase) from poplar that regulates tree stature. Plant Physiol.

[58] El-Sharkawy I, El Kayal W, Prasath D, Fernandez H, Bouzayen M, Svircev AM (2012). Identification and genetic characterization of a gibberellin 2-oxidase gene that controls tree stature and reproductive growth in plum. J Exp Bot.

[59] Yang J, Guo Z (2007). Cloning of a 9-cis-epoxycarotenoid dioxygenase gene (SgNCED1) from Stylosanthes guianensis and its expression in response to abiotic stresses. Plant Cell Rep.

[60] Bailey TL, Gribskov M (1998). Combining evidence using p-values: application to sequence homology searches. Bioinformatics.

[61] Livak KJ, Schmittgen TD (2001). Analysis of relative gene expression data using real-time quantitative PCR and the 2(−Delta Delta C(T)) method. Methods.

