# Supplementary figures and images for: Genome-wide identification and expression profiling reveal tissue-specific expression and differentially-regulated genes involved in gibberellin metabolism between Williams banana and its dwarf mutant
Source: BMC Plant Biol. 2016 May 27;16:123. doi: 10.1186/s12870-016-0809-1 (PMC4884393; doi:10.1186/s12870-016-0809-1)

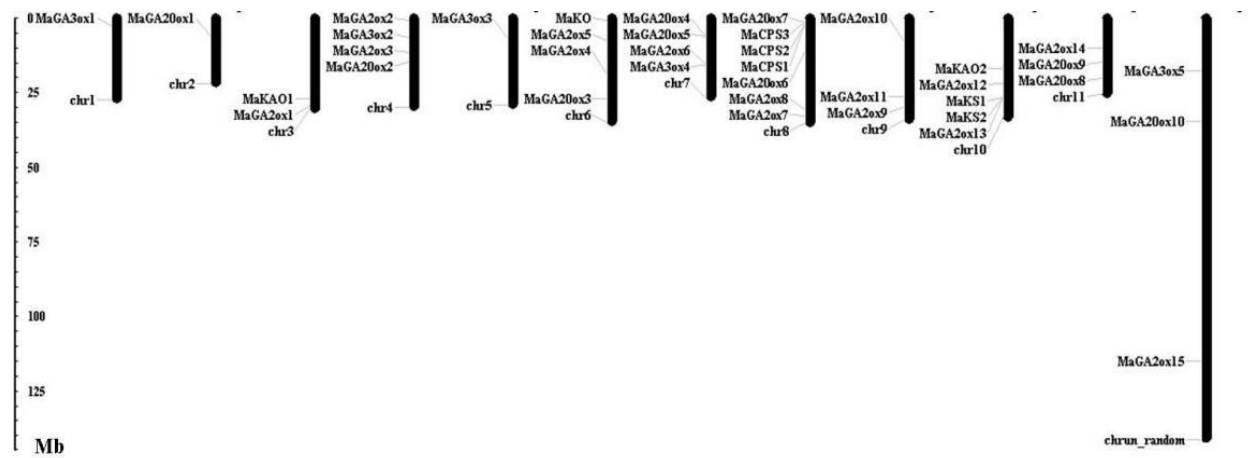

**Fig.S1.** Chromosomal locations of 38 banana candidate genes on the banana

A genome

Supplement: Additional file 1: Figure S1. — Chromosomal locations of 38 banana candidate genes on the banana A genome. (PDF 61 kb) [file 12870_2016_809_MOESM1_ESM.pdf]
