# Supplementary material for: Genome-wide identification and expression profiling reveal tissue-specific expression and differentially-regulated genes involved in gibberellin metabolism between Williams banana and its dwarf mutant
Source: BMC Plant Biol. 2016 May 27;16:123. doi: 10.1186/s12870-016-0809-1 (PMC4884393; doi:10.1186/s12870-016-0809-1)
Supplement: Additional file 2: Table S1. — The accession numbers of protein sequences cited in this study. (PDF 45 kb) [file 12870_2016_809_MOESM2_ESM.pdf]

Table S1. The accession numbers of protein sequences cited in this study

Gene name	Accession number
AtCPS	NP_192187
AtKO	NP_197962
AtKS	NP_178064
AtKAO1	NP_172008
AtKAO2	NP_001189657
AtGA20ox1	NP_194272
AtGA20ox2	NP_199994
AtGA20ox3	NP_196337
AtGA20ox4	NP_176294
AtGA20ox5	NP_175075
AtGA2ox1	CAB41007
AtGA2ox2	CAB41008
AtGA2ox3	CAB41009
AtGA2ox4	AAG51528
AtGA2ox6	AAG00891
AtGA2ox7	AAG50945
AtGA2ox8	CAB79120
AtGA3ox1	AAO64762
AtGA3ox2	NP_178150
AtGA3ox3	NP_193900
AtGA3ox4	NP_178149
OsCPS	BAS78107
OsKO	BAS98310
OsKS	BAS90962
OsKAO	BAS95781.1
OsGA20ox1	BAF13865
OsGA20ox2	BAJ09478
OsGA20ox3	BAF20901
OsGA20ox4	BAH93154
OsGA20ox5	XP_015628260
OsGA20ox6	XP_015633778
OsGA20ox7	XP_015648463
OsGA20ox8	XP_015635293
OsGA2ox1	BAB40934
OsGA2ox2	BAC16751
OsGA2ox3	BAC16752
OsGA2ox4	AAU03107
OsGA2ox5	BAF20604
OsGA2ox6	CAE03751
OsGA2ox7	XP_015633380
OsGA2ox8	XP_015638414
OsGA2ox9	XP_015624176
OsGA2ox10	AAT01379

OsGA2ox11	BAF14632
OsGA3ox1	NP_001054798
OsGA3ox2	NP_001042186
ZmCPS/AN1	NP_001105329.1
ZmKO	NP_001141872
ZmKS	XP_008668898.1
ZmKAO	NP_001105586
GmCPS1	XP_006604437.1
GmKO	XP_003543711.2
GmKS1	XP_006585394.1
GmKS2	XP_006585395.1
GmKAO	XP_003546291.1
