# Supplementary material for: Genome-wide identification and expression profiling reveal tissue-specific expression and differentially-regulated genes involved in gibberellin metabolism between Williams banana and its dwarf mutant
Source: BMC Plant Biol. 2016 May 27;16:123. doi: 10.1186/s12870-016-0809-1 (PMC4884393; doi:10.1186/s12870-016-0809-1)
Supplement: Additional file 3: Table S2. — Primer sequences of the reference gene and GA metabolism genes for qRT-PCR in this study. (PDF 71 kb) [file 12870_2016_809_MOESM3_ESM.pdf]

Table S2. Primer sequences of the reference gene and GA metabolism genes for qRT-PCR in this study

Gene	Entry name	Forward primer (5'→3')	Reverse primer (5'→3')
Actin	AB022041	GCCATACAGTGCCAATCTACGAGG	ATGTCACGAACAATTTCCCGCTCA
MaCPS3	GSMUA_Achr8T31530_001	AAAGCTGTGCAGGGAGGTT	CTTGGTGTGGTGGTTGAGG
MaKO1	GSMUA_Achr6T00910_001	GAGGAACCCGAAGAATGGA	ACGCAAGAAATCAGCATCG
MaKS1	GSMUA_Achr10T20910_001	GGTTGAGAAGTGGGATGGAA	ATGCAGATGCCCTAATACCG
MaKAO1	GSMUA_Achr3T27540_001	AGCAGGTTATGCCCTGGAA	CCATGCAATTGTCGGTAGG
MaGA20ox1	GSMUA_Achr2T01010_001	GAAGGAAACCCTCTCGTTCC	ATCCGAGCACCTCCATTATCT
MaGA20ox2	GSMUA_Achr4T16380_001	CCTCTCTGTCACAAGCTACGC	AAATCAGGGCCTAAGATGGAA
MaGA20ox3	GSMUA_Achr6T25910_001	ATGAGGTGCAACAGCTACCC	TCTTGTACCTCCCGTTGGAC
MaGA20ox4	GSMUA_Achr7T08230_001	GGCGGAGAACACCAAGAAT	TGGCCTGAGCGTACTCAAC
MaGA20ox5	GSMUA_Achr7T08240_001	ACTGGAAGGAGGTGTTCGACT	TTAAGCTGGTCTGGTCCTTGA
MaGA20ox6	GSMUA_Achr8T19120_001	CATCGTGGAGGACTACATCGT	CTCGAAGAACTCACGGAAGTG
MaGA20ox7	GSMUA_Achr8T32560_001	TGGCCCTCGATAAAGAGAGA	GTCTACGACAGGGGAGTCCA
MaGA20ox8	GSMUA_Achr11T11840_001	TGAGGATGGGAAGGGACTACT	CATGAAGGTGTCGCCTATGTT
MaGA20ox9	GSMUA_Achr11T18740_001	GCCACGGTTTCTTCCAACT	CCAGCACGGAGACGAAGTA
MaGA20ox10	GSMUA_AchrUn_randomT21840_001	TGGAAGGAGACCTTCACGTT	TTATCTGCAGCGACAGGTTG
MaGA3ox2	GSMUA_Achr4T08970_001	CATCTCCTGCTTCTTCTC	CTTCATCTCGTCGCTATA
MaGA3ox3	GSMUA_Achr5T09790_001	CGCCTTCTGTGACGTAATAG	GAGCATCATCCGCATCAA
MaGA3ox4	GSMUA_Achr7T13240_001	GATATGGCATCGCTAACA	CATCCAACTTGTGTATTCC
MaGA3ox5	GSMUA_AchrUn_randomT03870_001	CCTCCTTCTACTCCAATA	GCCTGTTATATTCTTCCATC
MaGA2ox1	GSMUA_Achr3T31410_001	AAGGTGCTTGAACTGATG	ATGGAGGATAGTGGTTGA
MaGA2ox3	GSMUA_Achr4T15110_001	ATACTTATCAGCCGTCAG	TGGAGGATAGTGATTGAG
MaGA2ox4	GSMUA_Achr6T21950_001	CGACAACAGGTAAAGATG	GGAAGACACAGGAATATG
MaGA2ox5	GSMUA_Achr6T26900_001	TTCAGCAGGTTAGTGATG	CTCCAGAATAGGGCATTT
MaGA2ox6	GSMUA_Achr7T13930_001	CATATCAGCAGTCAGGAA	CTCAAGACCGAATCACTC
MaGA2ox7	GSMUA_Achr8T03660_001	ATTCACACGGACCTAATC	ATGCTTCTATATGGAGAGG
MaGA2ox8	GSMUA_Achr8T27270_001	GTGTCCATGATCTACTTC	GTGAACTCCTTGTACTTG
MaGA2ox9	GSMUA_Achr9T06460_001	TTGAACTGATAGCCGAAG	GCATCTGGGATAGTGATT
MaGA2ox10	GSMUA_Achr9T11880_001	CAATACCTGCTCAAGTGT	TAACAGTGAACCATCTCC
MaGA2ox11	GSMUA_Achr9T21260_001	TCATCAGGATCAAGTAGG	CTTCGTATTGGTCATCAC
MaGA2ox12	GSMUA_Achr10T13090_001	TCCGAGTCATACAAGAGT	GAAGTAGCAGAGTGAGATT
MaGA2ox13	GSMUA_Achr10T21600_001	GTCGGTGATTCCTTACAG	CGAGACTTGAGACCATTG
MaGA2ox14	GSMUA_Achr11T14320_001	TACCTTCTTCTCCATTCC	CACTTGCTTCACATACTC
MaGA2ox15	GSMUA_AchrUn_randomT06450_001	GCCACATCTCTTCATCAG	ATAGCAACTACCCTTCTCT
